# Nitro-Heterocyclic compounds induce apoptosis-like effects in *Leishmania (L). amazonensis* promastigotes

**DOI:** 10.1590/1678-9199-JVATITD-1444-18

**Published:** 2019-03-11

**Authors:** Daiane Barros Dias Mendonça, Renata Ellen Costa Silva, Fanny Palace-Berl, Cleusa FH Takakura, Sandra Regina C Soares, Lucia Maria Almeida Braz, Leoberto Costa Tavares, Jose Angelo Lauletta Lindoso

**Affiliations:** 1 Laboratory of Serum Epidemiology, Faculty of Medicine, University of São Paulo, São Paulo, SP, Brazil; 2 Institute of Tropical Medicine, University of São Paulo, São Paulo, SP, Brazil; 3 Laboratory of Planning and Development of Pharmaceuticals, Department of Biochemical-Pharmaceutical Technology, Faculty of Pharmacy, University of São Paulo, São Paulo, SP, Brazil; 4 Department of Pathology, Faculty of Medicine, University of São Paulo, São Paulo, SP, Brazil; 5 Laboratory of Protozoology, Institute of Tropical Medicine, Faculty of Medicine, University of São Paulo, São Paulo, SP, Brazil; 6 Institute of Infectology Emilio Ribas, Secretary of State for Health, São Paulo, SP, Brazil; 7 Center for Tropical Medicine, Faculty of Medicine, University of Brasilia, Brasilia, Federal District, Brazil.

**Keywords:** Leishmaniasis treatment, Nitro-heterocyclic compounds, Leishmania (L.) amazonensis

## Abstract

**Background:**

Three drugs - pentavalent antimonials, amphotericin B and pentamidine - are currently used for leishmaniasis treatment. They are administered for long periods, only parenterally, and have high cardiac, renal and hepatic toxicities. Therefore, the investigation of new compounds is required. Nitro-heterocyclic derivatives have been used as possible drug candidates to treat diseases caused by trypanosomatids.

**Methods:**

*Leishmania (L.) amazonensis* promastigotes (MHO/BR/73/M2269), maintained in the Laboratório de Soroepidemiologia - Instituto de Medicina Tropical- USP, were exposed to five nitroheterocyclic derivatives, with differences at phenyl-ring position 4: BSF-C_4_H_9_, BSF-H, BSF-NO_2_, BSF-CH_3_ and BSF-Cl, for 48 hours. After analyzing viability (MTT assay), we evaluated cellular-morphology activity of compounds by transmission electron microscopy (TEM) and measurement of apoptosis (phosphatidylserine expression) by flow cytometry.

**Results:**

EC_50_ of amphotericin B and BSF-CH_3_ were 0.50 (M and 0.39 (M respective. Other nitro-heterocyclic compounds presented EC_50_ higher than amphotericin B. All compounds showed greater AV- and PI-positive expression than amphotericin B at 100 (M, except BSF-NO_2_. TEM showed complete nuclear disfigurement with 100 (M of BSF-NO_2_, 25 and 6.25 (M of BSF-H, and 6.25 (M BSF-Cl; presence of vesicles within the flagellar pocket with 25 (M BSF-H; alteration of the kinetoplast with 25 (M BSF-C_4_H_9_, 25 (M of BSF-H, 6.25 (M BSF-CH_3_ and 6.25 (M of BSF-Cl.

**Conclusions:**

Nitro-heterocyclic compounds have shown activity against promastigotes of *L. amazonensis,* at lower concentrations. However, improvement of compound scaffolds are needed to assist the elucidation of the mechanism of action and to achieve greater activity.

## Background

About 1 billion people are affected by one or more neglected diseases. These diseases are associated with malnutrition, poverty, population displacement, poor housing and lack of resources [1]. Leishmaniasis is a neglected tropical disease, endemic in 98 countries, with approximately one million individuals affected. Brazil reports approximately 26,000 new cases per year [2]. It may include visceral, cutaneous or mucosal clinical manifestations, directly associated with *Leishmania* species, which causes the infection [3]; [4]; [5]. American cutaneous leishmaniasis (ACL) is characterized by different types of tegumentary manifestations, and is caused by several *Leishmania* species. However, *Leishmania (L.) amazonesis* is one of main species causing tegumentary leishmaniasis in Brazil, and can produce diffuse cutaneous, mucocutaneous and cutaneous manifestations, which are the most common presentation of ACL [4]. Only three drugs, namely pentavalent antimonials, amphotericin B and pentamidine [6]; [7]; [8], are currently used in the treatment of leishmaniasis, even before miltefosine. In view of the long period of treatment, painful injections, appearance of resistance, high costs and side effects that include nephrotoxicity, myalgia, pancreatitis, and others [9], these treatment options are limited. Advances in drug research produce an imperfect framework, which is not democratic since they do not benefit all individuals requiring treatment [10]. In 29 years, only 1,556 new drugs have been released on the market for the treatment of tropical diseases, with the last one having been developed in 2004 [11]. The search for new compounds for leishmaniasis treatment is necessary and urgent, due to the high toxicity of the drugs currently employed to treat it. Nitro-heterocyclic compounds have shown a significant activity against *Trypanosoma cruzi* [12], [13], which, like *Leishmania*, is a genus from the family *Trypanosomatidae*. Furthermore, a previous publication from our group reported activity by nitro-heterocyclic compounds against *Leishmania (L.) infantum* [14]. Herein, the studied compounds were designed, by molecular modification, based on the nifuroxazide denominated 5-nitro-2-furfurylidene-4-hydroxy-benzohydrazide, an antimicrobial agent. Because of its oxidative stress-inducing ability [13], studies have shown that the nitro group at position 5 of the furan ring is essential for the performance of antiparasitic activity. Therefore, this study aimed to evaluate, *in vitro*, the effect of nitro-heterocyclic compounds on promastigote forms of *Leishmania (L.) amazonensis* by flow cytometry analysis to detect phosphatidylserine expression and also by transmission electron microscope (TEM) to analyze the ultrastructural modifications of promastigotes.

## Methods

### Animals

BALB/c mice, heterogenic, male, aged 40 to 60 days, were obtained from “Biotério da Faculdade de Medicina” (Animal Facility*) -* Universidade de São Paulo (São Paulo University)*,* were housed under controlled temperature and 12h/12h light cycle with food for Laboratory rodents (NUVITAL, BRAZIL) and water *ad libitum*. All procedures involving experimental animals were approved by the Ethics Committee (CEUA 0440/9) and following the guidelines of the Brazilian College for Experiments with Animal (COBEA-law 11.794/2008).

### Parasite culture

The infected mice by *L. (L.) amazonensis* promastigotes (MHO/BR/73/M2269) were euthanized through via intraperitoneal (I,p.) administration of xylazine/ketamine anesthetizing cocktail. After this, the paw was macerated and centrifuged at 1550 g for 10 min. The pellet obtained was resuspended in 199 Hanks culture medium (CULTILAB, BRAZIL), pH 7.0, enriched with 10% Fetal Bovine Serum (CRIPION), gentamicin and penicillin, maintained in a BOD greenhouse (Biochemical Oxygen Demand) at 26 ºC, and used in the experiment.

### Nitro-heterocyclic compounds

The five compounds, 4-R-substituted-N'-[(5-nitrofuran-2-yl)methylene]benzohydrazide (R = -H, - Cl, -NO_2_, -CH_3_, -C_4_H_9_), were obtained through the molecular modification of nifuroxazide, 4-Hydroxy-N'-[(5-nitrofuran-2-yl)methylene]benzohydrazide. These compounds, which were previously published by Tavares et al. [12]; [15]; [16], were synthesized and purified and their chemical structures elucidated by nuclear magnetic resonance of ^1^H and ^13^C and elemental analysis of H, N and C. 

Viability assay - MTT [3-(4,5-dimetiltiazol-2yl)-2,5-difenil tetrazolium bromide]: Five million (5x10^6^) promastigotes in stationary phase, diluted in RPMI medium without red phenol, were distributed in 96-well plates in triplicate. Compounds were dissolved in dimethyl sulfoxide PA (DMSO) and diluted in RPMI medium, without phenol, to obtain concentrations from 800 μM to 0.09 μM. The final concentration of DMSO in the medium used for dissolving the compounds did not affect parasite viability during the assay. As a negative control, high percentages of DMSO were used in the medium. As a drug control, promastigotes were incubated with amphotericin B at the same concentrations of the compounds. After 48 hours at 32°C, 20 μL of MTT (Sigma; 5 mg/mL in PBS) was added to the parasite culture with or without the compounds and incubated at 26°C for 4 hours. Subsequently, 50 μL of SDS 10% was added and incubated overnight at 26°C. The viability of promastigotes was determined in a microplate reader (Multiskan MCC / 340 - Brazil) at 570 nm wavelength. The EC_50_ levels of compounds and amphotericin B were calculated in comparison to positive control.

### Assay of Flow Cytometry (FC) for detection of Phosphatidylserine expression

To analyze the effect of compounds by flow cytometry (FC), concentrations of 100 μM, 25 μM and 6.25 μM were used. These concentrations were selected based on better results obtained by Petri e Silva et al., 2016 [14]. Phosphatidylserine externalization was detected by Annexin V - FITC method (BD, USA) according to the manufacturer’s instructions. Briefly, 10^6^ *L. amazonensis* promastigotes were incubated with nitro-heterocyclic compounds at three concentrations (100, 25 and 6.25 μM) for 48 hours at 32 °C and 5% of CO_2_. Parasites were washed with sterile PBS, and immediately resuspended in binding buffer (BB) [1X]. Five microliters of fluorochrome-conjugated Annexin V was added to each 100 μL of the cell suspension and incubated for 15 min at room temperature, protected from light. Next, cells were washed again and resuspended in 200 μL of BB. Five microliters of propidium iodide (PI) staining solution (Becton-Dickinson, USA) was added, and the samples were analyzed in a Fortessa SRL Flow Cytometer (Becton-Dickinson, USA). The positive control of phosphatidylserine detection was induced by camptothecin. Cells were gated on forward and side scatter signals to eliminate debris from analysis. All fluorescence parameters were recorded with logarithmic amplification. Analysis was performed on 10,000 gated events stored on FACS DIVA; data were analyzed using the software program FlowJo (Treestar, USA). Three independent experiments were conducted, and the results were analyzed as follows: Annexin V negative - PI negative, indicates the intact membrane plasmatic or live cells; 2) Annexin V positive - PI negative, indicates the cells initiated apoptosis and 3) Annexin V positive - PI positive, represents late apoptotic or necrotic cells. 

### Transmission Electron Microscopy (TEM): Analysis of the ultrastructural modifications of the promastigotes

TEM was employed to analyze the effect of compounds at the following concentrations: 100 μM, 25 μM and 6.25 μM. These concentrations were selected based on better results obtained by Petri e Silva et al., 2016 [14]. Promastigotes were seeded in 199 Hanks medium with each of the nitro-heterocyclic compounds (100 μM, 25 μM and 6.25 μM.) and amphotericin B for 48h, at 32°C in 5% CO_2_. After this, promastigotes were fixed overnight at 4 °C, with 2.5% glutaraldehyde, in 0.1 M cacodylate buffer, pH 7.2. Cells were washed in cacodylate buffer and postfixed with 1% OsO_4_, 0.8% potassium ferrocyanide and 5 mM CaCl_2_ in the same buffer for 1 h, at room temperature. Next, they were dehydrated in graded acetone, embedded in Epon (propylene oxide) 1:1 and then washed in the same buffer. Ultra-thin sections were mounted on 300- mesh grids, stained with uranyl acetate and lead citrate. After this, ultrastructural modifications of promastigotes were examined using TEM JEOL JEM 1400. 

### Statistical Analysis

Statistical analysis was performed using the sigmoidal dose-response curves with the software GraphPad Prism, version 5.0 for Windows to find the effective concentration of 50% (EC_50_). 

## Results

### Cytotoxicity analysis

Viability of *L. amazonensis* in the presence of nitro-heterocyclic compounds and amphotericin B is presented in Table 1. The respective EC_50_ of amphotericin B and BSF-CH_3_ were 0.50 μM and 0.39 μM. Other nitroheterocyclic compounds presented EC_50_ higher than amphotericin B. 

**Table 1:** Viability determination of *L. (L.) amazonensis* in presence of nitro-heterocyclic compounds and amphotericin B.

Compounds	EC_50_ (μM)	CI 95%
Amphotericin B	0.50	0.6 - 0.3
BSF-C_4_H_9_	0.82	1.2 - 0.5
BSF-CH_3_	0.39	0.5 - 0.2
BSF-Cl	1.2	1.4 - 0.98
BSF-H	1.2	1.45 - 1.13
BSF-NO_2_	0.84	0.99 - 0.70

EC_50_:50% of effective concentration, determined after 48 h of incubation; CI 95%: confidence interval of 95% .

### Assay of Flow Cytometry (FC) for detection of Phosphatidylserine expression

**Figure 1:**
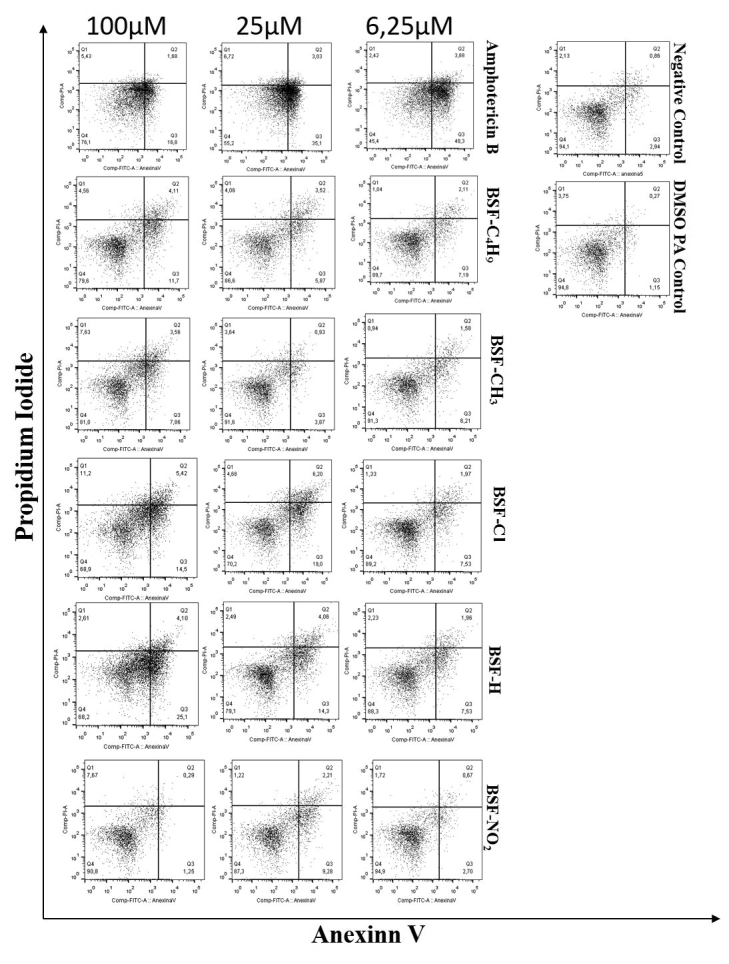
Promastigotes apoptosis determined by Annexin V-FITC staining after treatment with nitro-heterocyclic derivatives and amphotericin B. Flow cytometer analysis of the apoptotic and necrotic cells after incubation of 48 hours in presence of the substituted compounds. Negative Control (untreated) and DMSO PA control. Data are represented in dot-plot. Annexin V -FITC (X axis) and Propidium Iodide (PI) - PE (Y axis).

Each concentration of amphotericin B, and all nitro-compounds, except BSF-NO_2_ at 6.25 μM, presented a higher phosphatidylserine index in relation to the negative control. All compounds showed AV- and PI-positive expression in relation to amphotericin B at 100 μM, except BSF- NO_2_, which induced late apoptosis (Table 2 and Fig.1). The negative control showed PI fluorescence intensity of 2.94%. BSF- NO_2_ and BSF-CH_3_ compounds showed no significant difference from the negative control. 

**Table 2:** Detection of phosphatidylserine expression by Annexin V method on *L.(L) amazonensis* promatigotes exposed to different concentrations of nitro-heterocyclic derivatives and amphotericin B.

	PI+ (%)	**AV+ (%)**	**PI+ AV+ (%)**
Negative Control	2.13	2.94	0.86
DMSO PA Control	3.75	1.15	0.27
[100 µM]			
Amphotericin B	5.43	16.8	1.68
BSF-C_4_H_9_	4.56	11.7	4.11
BSF-CH_3_	7.63	7.86	3.56
BSF-Cl	11.2	14.5	5.42
BSF-H	2.61	25.1	4.1
BSF-NO_2_	7.67	1.25	0.29
[25 µM]			
Amphotericin B	6.72	35.1	3.03
BSF-C_4_H_9_	4.06	5.87	3.52
BSF-CH_3_	3.64	3.87	0.93
BSF-Cl	4.68	19	6.2
BSF-H	2.49	14.3	4.06
BSF-NO_2_	1.22	9.28	2.21
[6.25 µM]			
Amphotericin B	2.42	48.3	3.88
BSF-C_4_H_9_	1.04	7.19	2.11
BSF-CH_3_	0.94	6.21	1.58
BSF-Cl	1.33	7.53	1.97
BSF-H	2.23	7.53	1.96
BSF-NO_2_	1.72	2.7	0.67

Data expressed by the percentage of the average fluorescence intensity of Annexin V-FITC and Propidium Iodide-PE on *L.(L) amazonensis* promatigotes. Incubation for 48 hours, at 32 °C and 5% of CO_2_. AV+ (Q3): Annexin V positive; PI+ (Q1): Propidium iodide positive PI+ AV + (Q2): Annexin V and Propidium iodide positive; Negative control: promastigotes untreated.

### Transmission Electron Microscopy (TEM): Analysis of the ultrastructural modifications of the promastigotes

**Figure 2:**
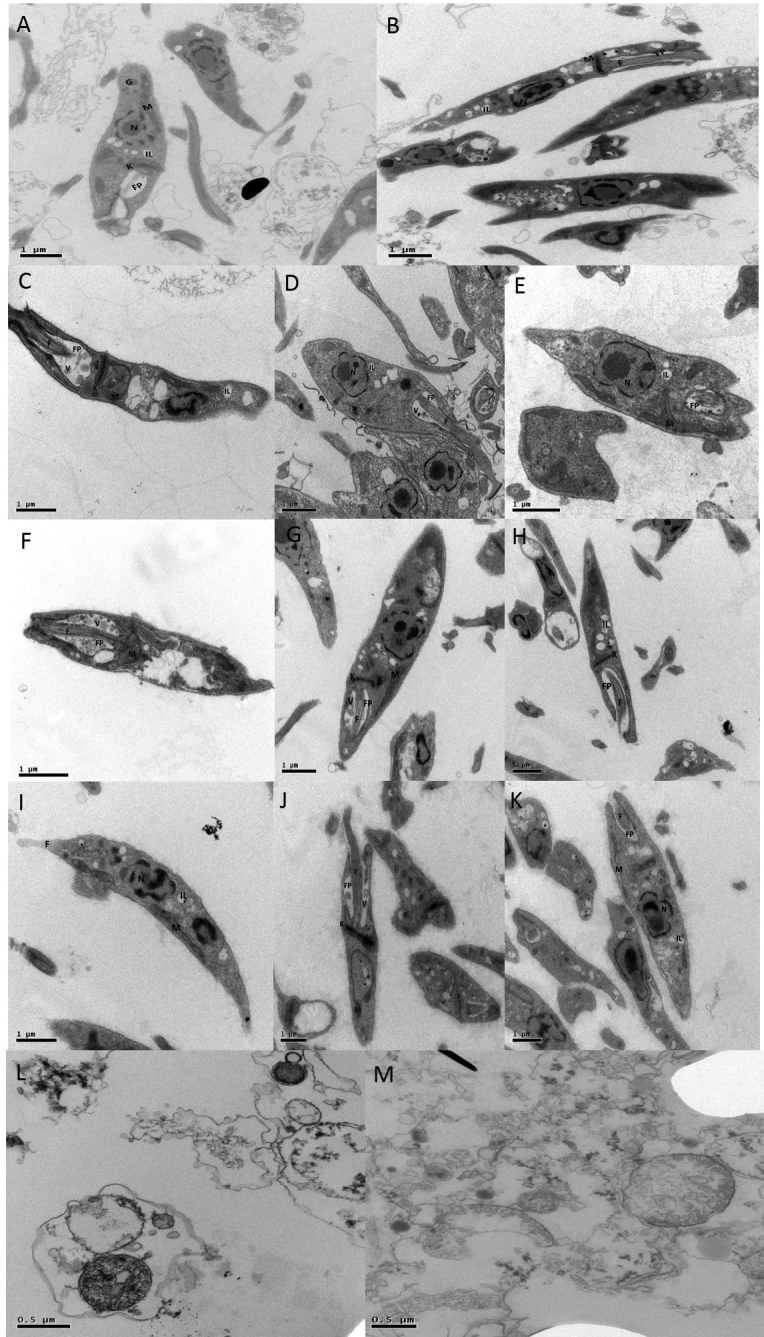
Promastigotes analyzed by TEM (Transmission Electron Microscopy) after exposure to nitro-heterocyclic compounds and controls. A/B - Negative control; C - BSF-NO_2_ 100 μM; D/E- BSF-C_4_H_9_ 25 μM; F - BSF-H 25 μM; G/H - BSF-H 6.25 μM; I - BSF-CH_3_ 6.25 μM; J/K - BSF-Cl 1.56 μM; L - Positive Control Amphotericin B 100 μM; M - Positive control Amphotericin B 25 μM. N - Nucleus; FP - Flagellar Pocket; K - Kinetoplast; V - Vesicles; IL - Inclusions Lipid; M - Mitochondria; G - Glicossome.

According to Figure 2 (A-B), untreated promastigotes showed elongated and preserved structure. All organelles from promastigotes treated with amphotericin B presented intense and important denaturation (Fig 2 L-M). The following conditions of promastigotes were observed according to concentrations of compounds: 1- mitochondrial swelling around the kinetoplast, retraction of flagella, presence of innumerous vacuoles in the flagellar pocket, nuclear disorganization and rounding of the parasite, when treated with 25 µM and 6.25 µM of BSF-H; 2- DNA decondensation promoted by modification of nuclear morphology with 6.25 µM of BSF-CH_3_; 3- vacuoles in the flagellar pocket, absence of chromatin and nuclear material, mitochondrial fragmentation and intense cytoplasmic alterations, but with elongated structure with 1.56 µM of BSF-Cl; 4- rounding of the parasite, mitochondrial fragmentation, nuclear disorganization and total or partial retraction of flagella, with 25 µM BSF-C_4_H_9_; and 5 - intense presence of vacuoles in the flagellar pocket and cytoplasm, mitochondrial swelling and nuclear alterations with BSF- NO_2_. By TEM, the promastigotes were analyzed using intermediate concentrations of the compounds. According to the results of FC (Figure 2) and MTT assay (Table 1) we observed the following: complete nuclear disfigurement with 100 μM of BSF-NO_2_ (C), 25 and 6.25 μM of BSF-H (F-G-H) and 6.25 μM BSF-CL (J); presence of vesicles within the flagellar pocket with 25 μM BSF-H (F) and alteration of the kinetoplast with 25 μM BSF-C_4_H_9_ (D-E), 25 μM of BSF-H (F), 6.25 μM BSF-CH_3_ (I) and 6.25 μM of BSF-Cl (K). Detachment of the nuclear membrane, chromatin condensation, loss of organelles in the cytoplasm and total parasite disfigurement were observed in parasites treated with amphotericin B. 

## Discussion

Although leishmaniasis is a disease of epidemiological relevance, it remains neglected and lacks an adequate treatment. Thirty to 50 percent of patients treated with the current drugs usually present with side effects such as tachycardia, skin lesions, headaches and vomiting. After continuous treatment, effects such as hypotension, hypoglycemia, cardiac changes, nephrotoxicity and sudden death have been described [4]; [8]. Considering these shortcomings in the treatments currently available for leishmaniasis, the search for new more effective compounds against the disease is fundamental. It should be noted that there are few new drugs on the world market for tropical diseases. It is known that of the 1,223 therapeutic chemicals introduced and marketed worldwide between 1975 and 1996, 379 are actually therapeutic innovations and less than 1% of these innovations were intended for tropical diseases including leishmaniasis [11]; [17]. Additionally, there is an increase of resistance to the drugs presently utilized to treat leishmaniasis. Drug combination is an interesting leishmanicidal chemotherapy and has shown some usefulness, since *Leishmania* spp has developed different immunomodulatory strategies that are essential for the establishment of the infection. Understanding the mechanisms associated with immune evasion and disease progression becomes essential for the development of novel therapies and vaccine approaches. Furthermore, more knowledge about public health policies to develop leishmanicidal drugs is also required.

Recently, research targeting the molecular and cellular machinery of the parasite, including using *in vitro* and *in vivo* studies has been conducted. Enzymes involved in synthesis of polyamines, including trypanothione reductase, are essential for the survival of *Leishmania* spp, justifying them as drug targets, such as proteases and arginase and transports via LdAAP3 and LdAAP7. A strategy to increase drug distribution through the liposomal intramononuclear phagocyte system has been explored [18]. Nitro-heterocyclic compounds are antimicrobial drugs [19] that have been shown to be a treatment option against trypanosomatids. Since the 1950s Evans Niemegeers and Pakchanian, have identified them as an alternative therapy for Chagas disease and sleeping sickness [20]. The action mechanism of these compounds is unclear; however, it is known to be based on inhibiting the mitochondrial respiration, lipid peroxidation, inactivation of peroxidases and glycolysis arginases [19].

In the current study *L. amazonensis* promastigotes were exposed to nitroheterocyclic compounds at different concentrations for 48 hours. Apoptosis was observed in 71.2% of the parasites exposed to the compound BSF-H and only in 62.3% of those exposed to amphotericin B, showing that the BSF-H can be as effective as amphotericin B. The MTT assay (viability) revealed that, when tested at low concentrations, the compounds proved to be good candidates for *in vivo* studies, presenting an EC_50_ up to 0.39 μM (BSF-CH_3_), more effective than the positive control, amphotericin B. Therefore, the compound was capable of inhibiting the growth of *Leishmania* at lower concentrations. Evaluation of phosphatidylserine externalization [21] was used for cellular apoptosis analysis. Phosphatidylserine is a phospholipid located in the interior of plasma membrane that shifts to the surface of the cellular membrane, through breakage of this membrane in apoptotic cells. Annexin V is a binding protein dependent on phospholipid calcium that binds preferentially to phosphatidylserine [22]. In the current study Annexin V was employed to evaluate the externalization of phosphatidylserine. Changes in cell membrane integrity were analyzed by PI staining, characterized by intercalating the DNA of parasites with a compromised membrane, alterations not presented by live parasites with an intact membrane [21]; [22] [23]. In the present study, all compounds showed greater AV- and PI-positive expression in relation to amphotericin B at 100 μM, except BSF-NO_2_. The parasites treated with higher concentrations of BSF-Cl and BSF-H presented apoptosis when analyzed by flow cytometry, which did not occur when they were treated with the higher concentrations of BSF-C_4_H_9_, BSF- NO_2_ and BSF-CH_3_. With these compounds, parasites entered apoptosis at lower concentrations. Transmission electron microscopy (TEM), an excellent assay for the evaluation of morphological changes of the parasites [24], was utilized to compare the morphology of viable parasites (negative control) versus those treated with the nitro-compounds. Usually promastigotes present elongated fusiform morphology with a nucleus and highly defined kinetoplast. However, the parasites treated with these compounds presented morphological changes such as formation of vesicles inside the flagellar pocket, disfigurement of the nucleus and shrinkage of parasites. Such characteristics have already been described by Rodrigues et al., 2008 [25]; Medeiros et al., 2011 [26] and Rottini et al., 2015 [27] in studies of compounds with possible antileishmania activity. In this study, TEM showed complete nuclear disfigurement after administration of 100 μM of BSF- NO_2_, 25 and 6.25 μM of BSF-H and 6.25 μM BSF-Cl; presence of vesicles within the flagellar pocket at 25 μM BSF-H; alteration of the kinetoplast after 25 μM BSF-C_4_H_9_, 25 μM of BSF-H, 6.25 μM BSF-CH_3_ and 6.25 μM of BSF-Cl. 

The nitro-heterocyclic compounds have shown effective *in vitro* activity, by inhibiting the growth of *T. cruzi* epimastigotes and amastigotes [12]. This result suggested that a similar activity could be found against *Leishmania*, since both parasites belong to the family Trypanosomatidae, as found against *Leishmania* (*L.*) *infantum* [14]. Nevertheless, despite the similarity between these protozoa, it is believed that the molecular modification at position 4 of the phenyl ring of the compounds can result in significant changes of molecular properties and trigger distinct responses in each protozoan. These observations suggested that further analysis including more substitutions in nitro- compounds may help elucidate activity against *Leishmania* in comparison to *T. cruzi*.

## Conclusions

 All compounds analyzed show *in vitro* activity against *Leishmania (L.) amazonensis* promastigotes, especially those compounds at lower concentrations. Further investigations are required to determine the mechanism of the nitro-heterocyclic action in *Leishmania*. 

## Abbreviations

Not applicable.
